# Cytokine Release Assays as Tests for Exposure to *Leishmania*, and for Confirming Cure from Leishmaniasis, in Solid Organ Transplant Recipients

**DOI:** 10.1371/journal.pntd.0004179

**Published:** 2015-10-23

**Authors:** Eugenia Carrillo, Nerea Carrasco-Antón, Francisco López-Medrano, Efrén Salto, Laura Fernández, Juan Víctor San Martín, Jorge Alvar, Jose María Aguado, Javier Moreno

**Affiliations:** 1 WHO Collaborating Centre for Leishmaniasis, Centro Nacional de Microbiologia, Instituto de Salud Carlos III, Madrid, Spain; 2 Unidad de Enfermedades Infecciosas, Hospital Universitario 12 de Octubre, Universidad Complutense, Madrid, Spain; 3 Servicio de Microbiología, Hospital Universitario 12 de Octubre, Universidad Complutense, Madrid, Spain; 4 Servicio de Medicina Interna, Hospital de Fuenlabrada, Madrid, Spain; 5 Head of Visceral Leishmaniasis Program, Drugs for Neglected Diseases initiative (DNDi), Geneva, Switzerland; Addis Ababa University, ETHIOPIA

## Abstract

Spain has one of the world’s largest pools of organ donors and is a global leader in terms of the number of transplants it performs. The current outbreak of leishmaniasis in Fuenlabrada (in the southwest of the region of Madrid, Spain) has involved 600 clinical cases since late 2009 (prevalence 0.2%). It may therefore be wise to monitor the town’s transplanted population for *Leishmania infantum*; its members are immunosuppressed and at greater risk of infection and relapse following treatment. The present work examines the use of cytokine release assays to determine the prevalence of *Leishmania* infection in this population, and to confirm recovery following treatment for visceral leishmaniasis (VL). The humoral and cellular immune responses to *L*. *infantum* were characterized in 63 solid organ transplant (SOT) recipients from Fuenlabrada, 57 of whom reported no previous episode of VL (NVL subjects), and six of whom had been cured of VL (CVL subjects). Seventeen subjects (12 NVL and 5 CVL) showed a patent lymphoproliferative response to soluble *Leishmania* antigen (SLA). Stimulation of peripheral blood mononuclear cell cultures and of whole blood with SLA led to the production of different combinations of cytokines that might serve to confirm *Leishmania* infection or recovery from VL and help prevent cured patients from relapsing into this serious condition.

## Author Summary

We have used cytokine release assays to determine the prevalence of *Leishmania infantum* infection in solid organ transplant (SOT) recipients living in an area where the organism is endemic following an outbreak. Some 21.05% of SOT recipients with no previous history of leishmaniasis had been in contact with the parasite; the risk of these individuals becoming infected by *Leishmania* is high, a consequence of their need to be maintained in an immunosuppressed state. The results indicate the usefulness of whole blood stimulation assays, and of IFN-γ/TNF-α analysis, for determining exposure to *Leishmania* and confirming cure from visceral leishmaniasis in SOT recipients.

## Introduction

In Spain, leishmaniasis is an endemic zoonosis caused by *Leishmania infantum*. The national mean annual incidence is 0.45 cases/100,000 inhabitants, similar to that traditionally reported for the capital, Madrid (0.5 cases/100,000 inhabitants) [1]. A few years ago, an unusual, large outbreak of leishmaniasis was noted in the southwest of the Region of Madrid, and disease incidence in the town of Fuenlabrada is currently 21.54/100,000. Since 2009, more than 100 cases of visceral leishmaniasis (VL) have been recorded every year in Fuenlabrada; similar figures are expected for the coming years [2].

Immunosuppression is a major risk factor in the appearance of overt clinical leishmaniasis; it can also alter the presentation of the disease and response to treatment [3]. Indeed, the worldwide number of cases of VL in recipients of solid organ transplants (SOT) has quadrupled since the 1990s [4] (77% of those affected received a kidney transplant, the most commonly performed transplant operation). Most of these cases occurred in the Mediterranean basin, particularly in Spain, which has one of the world’s largest pool of organ donors and is among the foremost in performing SOTs.

Very little is known, however, about the relationship between leishmaniasis and transplants, and the prevalence of asymptomatic leishmaniasis is unknown [5]. During the recent outbreak in Fuenlabrada, eight out of the total 130 SOT recipients residing in the area developed VL (6.15%); the risk for these patients is therefore around 30 times that faced by immunocompetent individuals.

Recently, molecular and serological analyses of liver transplant recipients in an area of Brazil where leishmaniasis is endemic revealed a high prevalence of asymptomatic infection [6]. However, measurement of the cell-mediated immune response is critical when estimating exposure to pathogens causing intracellular infections. Immunity to leishmaniasis is associated with a strong Th1-type immune response, as demonstrated by a positive delayed type hypersensitivity (DTH) reaction in the Leishmanin skin test (LST), along with lymphocyte proliferation and the production of high levels of IFN-γ and TNF-α [7] following stimulation with soluble leishmania antigen (SLA).

T-cell-based interferon-γ (IFN-γ) release assays have been developed as alternatives to DTH tests [8]. For example, the whole-blood gamma interferon release assay (IGRA) has recently been reported capable of detecting subclinical infection in healthy individuals living in an area where VL is endemic [9]. Although some studies have been published on the use of IGRA in tuberculosis and cytomegalovirus infection [10, 11], the literature contains nothing on its use to detect *Leishmania* infection in SOT recipients. The aim of the present work was to test cytokine release assays as a means of determining the prevalence of *Leishmania* infection in SOT recipients, and to confirm recovery following treatment for VL. Assessing the exposure to *Leishmania* and the immunological memory of SOT recipients living in an area highly endemic for leishmaniasis should throw light on the infection rate in this population, help prevent those treated for VL from relapsing, and reveal the epidemiological features of this disease in the immunosuppressed within the context of an outbreak.

## Materials and Methods

### Population

Sixty three SOT (kidney, liver and heart) recipients were enrolled in the present study. All were aged 18 years or older, had undergone transplant surgery between 2005 and 2013 at the *12 de Octubre* University Hospital, and resided in the town of Fuenlabrada. Fifty seven subjects had experienced no previous episode of VL or compatible symptomology (NVL subjects), and six had been cured of visceral leishmaniasis (CVL subjects).

### Ethics statement

Recruitment and sample collection were performed in accordance with Good Clinical Practice guidelines. The study was approved by the ethics Committee of the *12 de Octubre* University Hospital. All subjects gave their written informed consent to be included in the study.

### Immunosuppressive treatment of SOT subjects

Recipients of a graft from a non-heart beating donor (30% of all SOT recipients) underwent induction therapy with intravenous (IV) rabbit anti-thymocyte globulin (ATG-Fresenius) (1.25 mg/kg daily for 5–7 days) and a calcineurin inhibitor (CNI) from day 6. Patients at high immunological risk received induction therapy with ATG for 1–3 days plus CNI from day 0. Basiliximab (20 mg on days 0 and 4) was provided to patients at high risk of CNI-related nephrotoxicity owing to advanced age or pre-transplant comorbidities. Immunosuppression was maintained with tacrolimus (0.1 mg/kg daily), mycophenolate mofetil (500–1000 mg twice daily) or mycophenolic acid (360 mg twice daily), and prednisone (0.5 mg/kg daily with progressive tapering beyond day 20 or 30). Perioperative prophylaxis consisted of a single dose of 2 g of IV cefazolin. Trimethoprim–sulphamethoxazole (160/800 mg 3 times weekly) or monthly IV pentamidine was provided as prophylaxis for *Pneumocystis jirovecii* pneumonia for the first nine months. Patients at high risk of cytomegalovirus disease were administered IV gancyclovir (5 mg/kg daily) or oral valgancyclovir (900 mg daily) for the first three months.

### Preparation of soluble *L*. *infantum* antigen for stimulation

*Leishmania infantum* antigen extract was prepared from promastigote stationary phase parasite cultures (JPC strain, MCAN/ES/98/LLM-722). SLA was obtained from parasites by washing in 1x phosphate-buffered saline (PBS) and centrifuging at 1000 *g* for 20 min at 4°C. The supernatant was removed and the pellet resuspended in lysis buffer (50 mM Tris/5 mM EDTA/HCl, pH 7; 1 ml for every 10^9^ parasites). The latter was then subjected to three rapid freeze/thaw cycles followed by three 20 s 40W pulses with a sonicator, and centrifuged at 27,000 *g* for 20 min at 4°C. The supernatants were then collected, aliquoted and stored at -80°C until use. Protein quantification was performed using the Bradford method employing the Bio-Rad Protein Assay kit (Bio-Rad, USA).

### Culture and stimulation of peripheral blood mononuclear cells

Peripheral blood mononuclear cells (PBMCs) were isolated by density centrifugation through Ficoll-Hypaque (Rafer, Spain). The collected cells were cultured in RPMI 1640 supplemented with 10% heat-inactivated foetal bovine serum, 100 IU/ml penicillin, 100 μg/ml streptomycin, 2 mM L-glutamine, 50 μM 2-mercaptoethanol, and 1 mM sodium pyruvate. They were then plated in 96 well-plates and kept with RPMI 1640 medium alone (unstimulated) or in RPMI 1640 supplemented with 10 μg/ml phytohaemagglutinin type M (PHAM) (Sigma-Aldrich, USA) (positive control), or with SLA (10 μg/ml). All were kept in a humidified, 5% CO_2_ atmosphere at 37°C for 5 days. Cell proliferation was measured by bromodeoxyuridine incorporation using the Cell Proliferation Biotrak ELISA kit (General Electric Healthcare Life Sciences, UK). The results are shown as a stimulation index (SI) for each mitogen or antigen. The cut-off for positive lymphoproliferation was calculated as the mean+3 SD (standard deviation) of the SI for 45 *Leishmania*-exposed but completely negative SOT subjects: SI = 2.76. The supernatants of the *in vitro* cell cultures were collected and stored at -20°C for cytokine quantification.

### Whole blood stimulation

Blood samples (9–10 ml) were collected from patients in tubes containing heparin. Aliquots (500 μl) of whole blood were incubated in tubes with 10 μg/ml SLA or 5 μg/ml PHAM as a positive control. An additional tube for which no stimulation was provided was included as a negative control. After adding the antigens and mixing thoroughly, the tubes were incubated at 37°C for 24 h. After centrifugation at 2000 *g* for 10 min, the supernatant plasma were collected and stored at -20°C for cytokine analysis.

### Cytometric quantification

IFN-γ, granzyme B, tumour necrosis factor-alpha (TNF-α), interleukin (IL)- 10 (IL-10), IL, 5, IL-17A, IL-2 and IL-4 were quantified in 50 μl of plasma and PBMC culture supernatants following PHAM (10 μg/ml, 120 h) or SLA stimulation (10 μg/ml, 24 h), using the BD Cytometric Bead Array Human Flex Set (Beckton Dickinson Biosciences, USA) following the manufacturer’s instructions. Data were acquired using a FACSCalibur flow cytometer and analysed using the Flow Cytometric Analysis Program Array (Beckton Dickinson Biosciences, USA).

### Antibody detection methods

An enzyme-linked immunosorbent assay (ELISA) was used to detect antibodies to SLA. Briefly, 96-well plates (NuncMaxisorp Immuno Plates, USA) were coated with 100 μl/well of 10 μg/ml SLA and left overnight at 4°C. The plates were then washed three times with PBS, 0.1% Tween 20 (PBS-T), pH7.4 and blocked with 200 μl/well of PBS containing 0.1% Tween 20 and 3% BSA for 1 h at 37°C. After washing with PBS-T, diluted blood plasma (1/200 in PBS-T) was added (100 μl/well) and incubated for 2 h at 37°C. The plates were then washed with PBS-T and 100 μl/well of 1/5000-diluted HRP-conjugated anti-human Ig (Invitrogen, USA) were added for 30 min at 37°C. All plates were then developed with 100 μl/well of Sigma Fast o-phenylene diamine dihydrochloride (OPD) tablets (Sigma, USA) for 20 min. The reaction was stopped with 50 μl/well of 2N HCl, and absorbance measured at 492 nm. All tests were performed in duplicate and the mean value recorded. The cut-off for seropositivity was calculated as described above at 0.132.

Immunofluorescent antibody titre (IFAT) analyses of plasma samples were performed using 2 × 10^5^ *L*. *infantum* promastigotes in PBS per well (MCAN/ES/98/LLM-722). Subject plasma was assayed as two-fold serial dilutions from 1/20 to 1/640 in PBS to determine total IgG levels using fluorescein isothiocyanate-conjugated goat anti-human IgG (Fluoline G) (BioMérieux, France) diluted 1/200. The threshold titre for positivity was set at 1:80.

Finally, rK39-ICT dipsticks were purchased from Leti Laboratories and the corresponding test performed following the manufacturer’s instructions.

### DNA isolation and real time PCR

DNA isolation and real time PCR (qPCR) were performed as described by Cunha et al. [12]. Briefly, DNA was extracted from 100 μL of peripheral blood by conventional phenol-chloroform extraction and eluted in 100 μl sterile distilled water. For qPCR, 1000 nM of R223 and 500 nM of R333 primers (Sigma-Aldrich, USA) for the small subunit rRNA (SSUrRNA) sequence were used [13]. Total DNA was used as a template in touchdown qPCR reactions involving the LightCycler FastStart DNA Master SYBR Green I kit (Roche Applied Science, Switzerland) [14].

### NNN culture of PBMCs

PBMCs (2x10^5^) from all SOT recipients were cultured in Novy-MacNeal-Nicolle medium. The presence/absence of *Leishmania* was checked every week up to 4 weeks.

### Statistical analysis

Immunological data were tested for normality using the Shapiro-Wilk test. Means were compared using the Mann-Whitney U test. The Spearman's rank test was used to seek correlations between immunological variables. Significance was set at p<0.05. All calculations were performed using GraphPad Prism 5.0 software (GraphPad Software, USA).

## Results

### Immunological response and parasite load

The *in vitro* cell proliferation assay with PHAM stimulation revealed the functional capacity of the PBMCs to mount a response in all immunosuppressed individuals.

**Fig 1:**
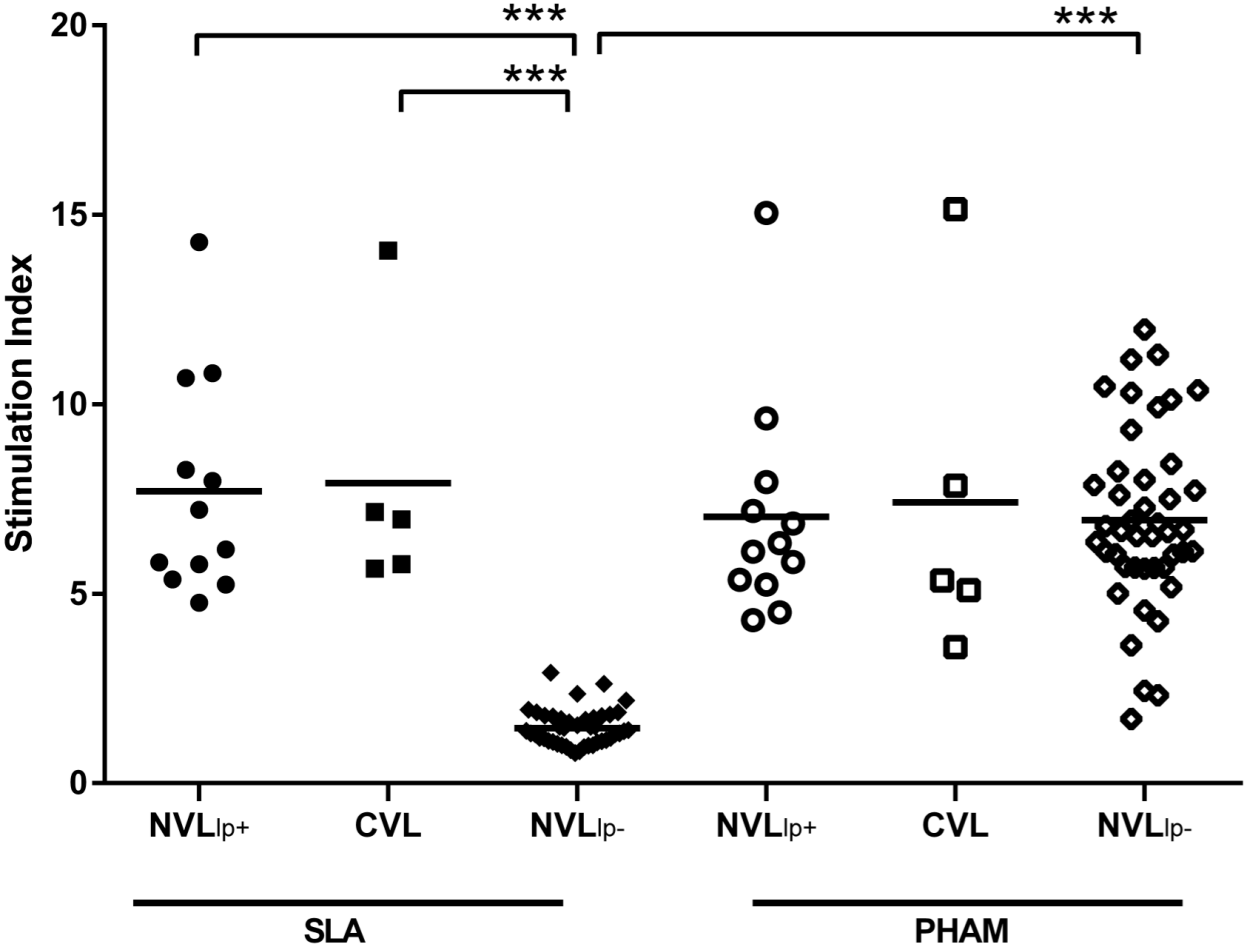
Lymphoproliferative response to SLA of PBMCs from solid organ (SOT) recipients—57 with no previous history of leishmaniasis (NVL) and 5 cured SOT recipients (CVL). NVL SOT recipients were then classified as either positive (NVL_lp+_) or negative (NVL_lp-_). ***p<0.001.

Some 21.05% of the NVL subjects (12/57) mounted a positive lymphoproliferative response to SLA stimulation (Fig 1) (hereinafter referred to as NVLpl+ subjects). The IFAT results revealed just two of these same 57 subjects to have mounted a positive serological response; the ELISA and rK39-ICT tests were positive in one of these latter two subjects. This person (i.e., positive in all three serological tests) also showed a strong lymphoproliferative response to SLA (SI ≥14). The subject who was serologically positive by IFAT alone showed no lymphoproliferative response and developed VL within a few days of testing (treatment was provided within 2 weeks of the original blood sampling), and for that reason was excluded for further analysis (S1 Fig).

The SLA-stimulated PBMC cultures returned positive results for five out of six CVL subjects. IFAT detected anti-*Leishmania* antibodies in two of these subjects, while the ELISA and rK39-ICT tests detected the same in only one. (Note: The single CVL subject who showed no lymphoproliferative response showed no cytokines in the supernatants of SLA-stimulated PBMC cultures or plasma [see below], and produced no antibodies against *Leishmania*. This subject relapsed four months after sampling and was therefore considered not cured, and excluded from further analysis).

No *Leishmania* DNA was detected in any blood sample from any SOT recipient. Absence of residual parasite DNA could be due to the reduced sample size, endemicity of the area, PCR sensitivity or episodic presence of parasite in blood. Neither were any NNN cultures positive for parasites.

### Cytokine profile supernatants of stimulated PBMC culture

**Fig 2:**
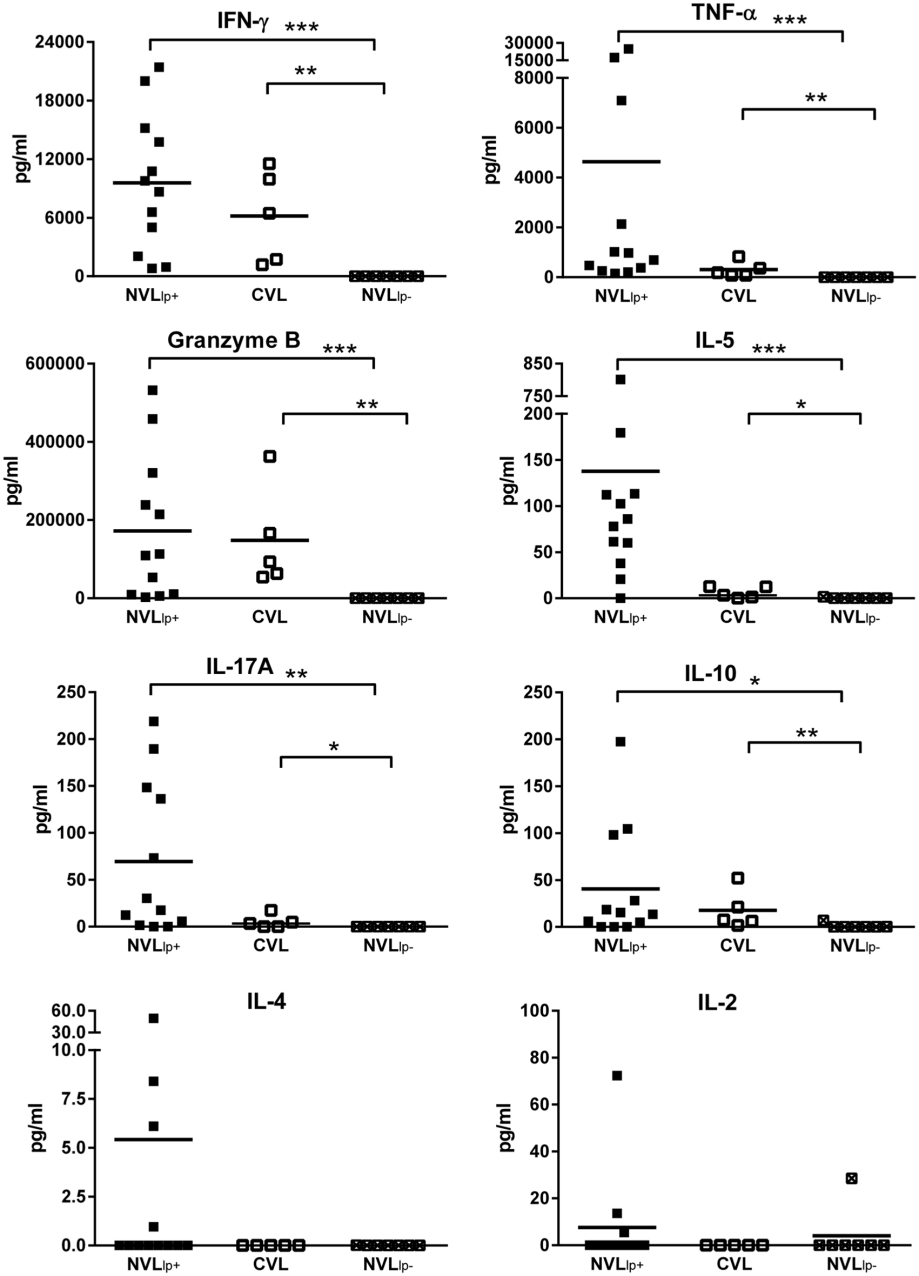
Cytokine and granzyme B production after PBMC stimulation with SLA for 5 days. IFN-γ, granzyme B, TNF-α, IL-10, IL-5, IL-17A, IL-2 and IL-4 concentrations (pg/ml) were measured in culture supernatants of 12 NVL_lp+_ subjects, 7 NVL_lp-_ subjects, and 5 CVL subjects. *p<0.05, **p<0.01, ***p<0.001.

The supernatants of *in vitro* SLA-stimulated cultures of PBMCs from NVL_lp+_ subjects showed significantly more IFN-γ (P<0.0001), granzyme B (P<0.0001), TNF-α (P<0.0001), IL-5 (P = 0.0004), IL-10 (P = 0.0413) and IL-17A (P = 0.0030) than those of NVL subjects who did not respond to SLA (hereinafter referred to as NVL_lp-_ subjects) (Fig 2). The supernatants of CVL subjects showed the same cytokine pattern as those of the NVL_lp+_ subjects: IFN-γ (P = 0.0013 compared to the NVL_lp-_ subjects), granzyme B (P = 0.0025), TNF-α (P = 0.0025), IL-5 (P = 0.0455), IL-10 (P = 0.0088), and IL-17A (P = 0.0379). No significant differences were found in the production of IFN-γ, granzyme B, IL-10, IL-17A, IL-4 and IL-2 between NVL_lp+_ and CVL subjects, while TNF-α (P = 0.036) and IL-5 (P = 0.0040) were higher in NVL_lp+_ subjects.

**Fig 3:**
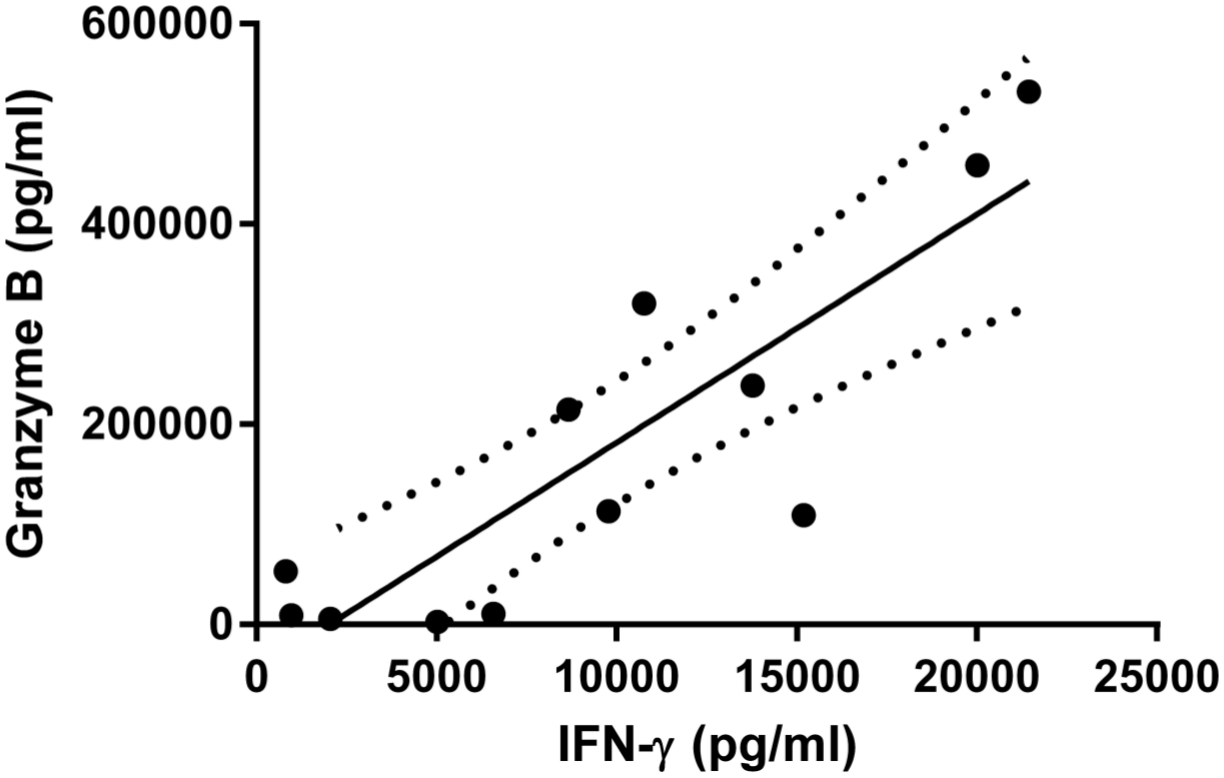
Linear regression for granzyme B and IFN-γ in SLA-stimulated PBMCs from NVL_lp+_ subjects. A significant correlation was found between these variables (r = 0.818, P = 0.00093).

In the 12 NVL_lp+_ subjects, IFN-γ production correlated with that of TNF-α (r = 0.853, P = 0.004), granzyme B (r = 0.818, P = 0.00093) and IL-10 (r = 0.810, P = 0.0012). By way of example, Fig 3 shows the correlation between IFN-γ and granzyme B.

Non-specific stimulation of the PBMCs with PHAM led to an efficient Th1 response that was similar in the NVL_lp+_, NVL_lp-_ and CVL subjects (Table 1). IL-10 production was however, significantly higher in the NVL_lp+_ subjects than in the NVL_lp-_ subjects (P = 0.0097).

**Table 1:** Cytokine and granzyme B production after PBMC stimulation with PHAM for 5 days. IFN-γ, granzyme B, TNF-α, IL-10, IL-5, IL-17A, IL-2 and IL-4 concentrations (pg/ml) were measured in culture supernatants of 12 NVL_lp+_ subjects, 7 NVL_lp-_ subjects, and 5 CVL subjects.

SOT	IFN-γ (pg/ml)	TNF-α (pg/ml)	IL-10 (pg/ml)	Granzyme B (pg/ml)	IL-17A (pg/ml)	IL-2(pg/ml)	IL-4 (pg/ml)	IL-5 (pg/ml)
	Mean SD	Mean SD	Mean SD	Mean SD	Mean SD	Mean SD	Mean SD	Mean SD
NVL_lp+_	41537.80 ± 49615	849.40 ± 1242	716.12** ± 548	559998.34 ± 1144904	635.11 ± 1117	41.08 ± 65	0.29 ± 0	80.96 ± 157
CVL	43593.00 ± 74291	973.40 ± 1269	203.50 ± 189	676302.00 ± 1456000	55.22 ± 89	11.74 ± 26	0.00 ± 0	1026.00 ± 2259
NVL_lp-_	9140.39 ± 9447	309.07 ± 230	235.64 ± 147	50851.16 ± 66809	56.19 ± 86	0.00 ± 0	0.00 ± 0	10.60 ± 26

Abbreviations:

NVL subjects: Solid organ transplant recipients whom reported no previous episode of VL or compatible symptomology;

NVL_lp+_: NVL subjects who mounted a positive lymphoproliferative response to SLA stimulation;

CVL: Solid organ transplant recipients cured form visceral leishmaniasis;

NVL_lp-_: NVL subjects who did not respond to SLA;

**p<0.01

### Quantification of cytokines in plasma from stimulated whole blood

**Fig 4:**
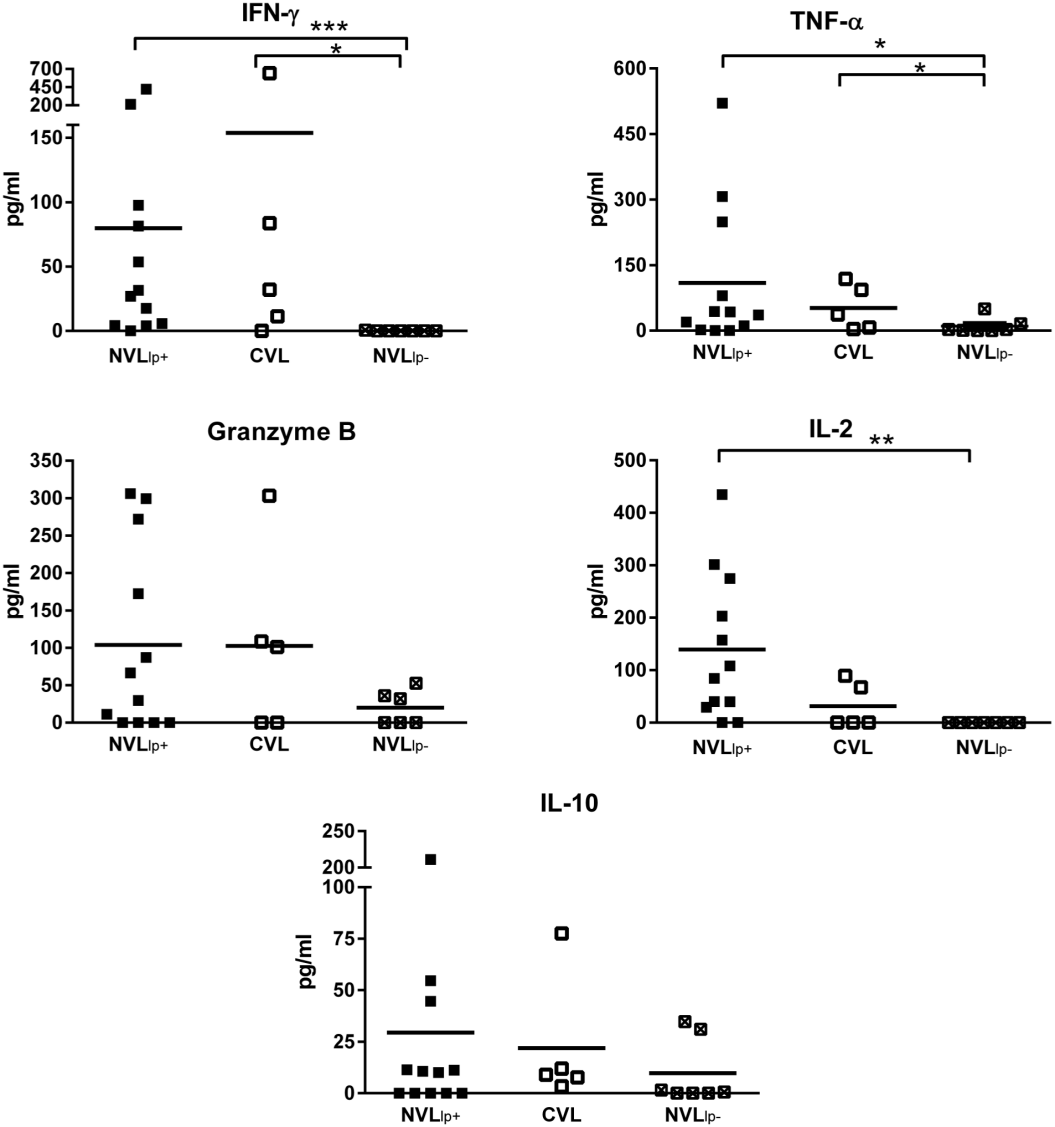
Cytokine and granzyme B production in whole blood plasma in response to SLA stimulation for 24 h. The IFN-γ, granzyme B, TNF-α, IL-10 and IL-2 concentrations (pg/ml) were measured for 12 NVL_lp+_ subjects, 7 NVL_lp-_ subjects, and 5 CVL subjects. *p<0.05, **p<0.01, ***p<0.001.

**Fig 5:**
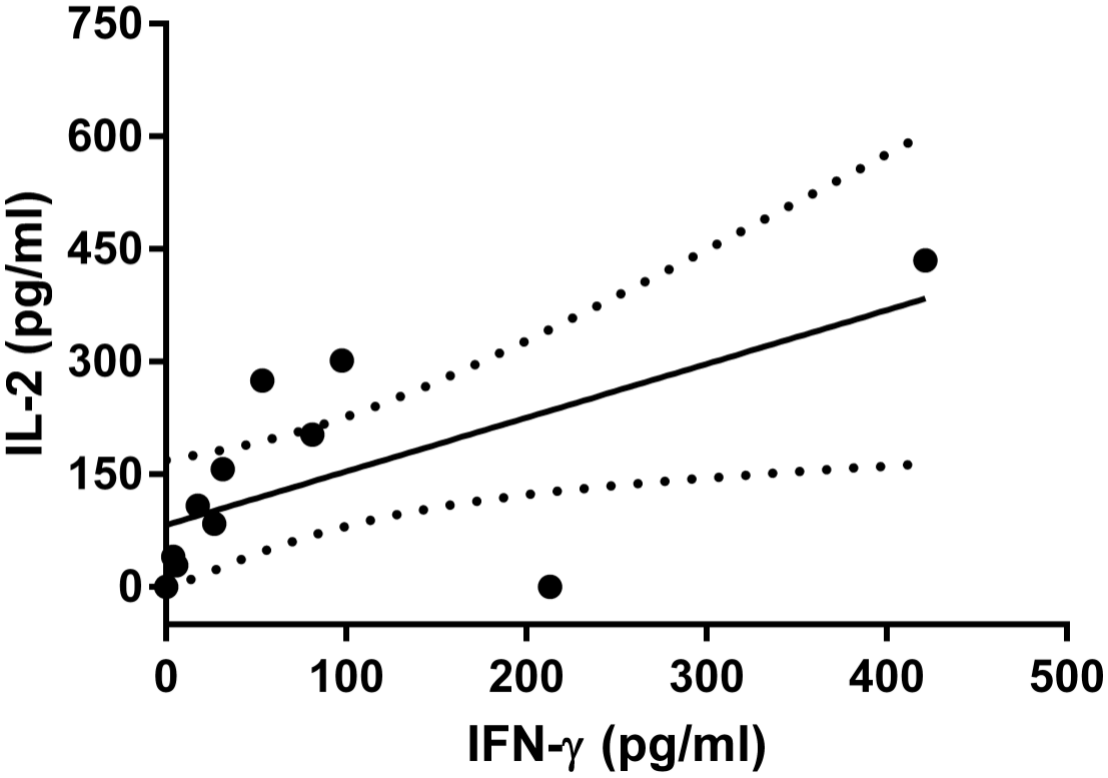
Linear regression of IFN-γ and IL-2 responses in plasma from SLA-stimulated whole blood plasma in NVL_lp+_ subjects. A significant correlation was detected between these variables (r = 0.616, P = 0.018).

SLA-stimulated whole blood from NVL_lp+_ subjects produced significantly more IFN-γ (P = 0.0004), TNF-α (P = 0.0469), and IL-2 (P = 0.0028) than that of SLA-stimulated whole blood from NVL_lp-_ subjects (Fig 4). In these NVL_lp+_ subjects, IFN-γ production correlated with IL-2 (r = 0.616, P = 0.018) (Fig 5). Similarly, CVL subjects with a positive lymphoproliferative response showed significantly higher concentrations of IFN-γ and TNF-α in plasma (P = 0.0101 and P = 0.0467 respectively) than the NVL_lp-_ subjects. No differences in IL-10 concentration were seen between the three different groups of subjects (Fig 4). Stimulating whole blood with SLA for 24 h did not induce production of IL-17A, IL-5 or IL-4 (S1 Table). No significant differences were found between NVL_lp+_ and CVL subjects.

**Fig 6:**
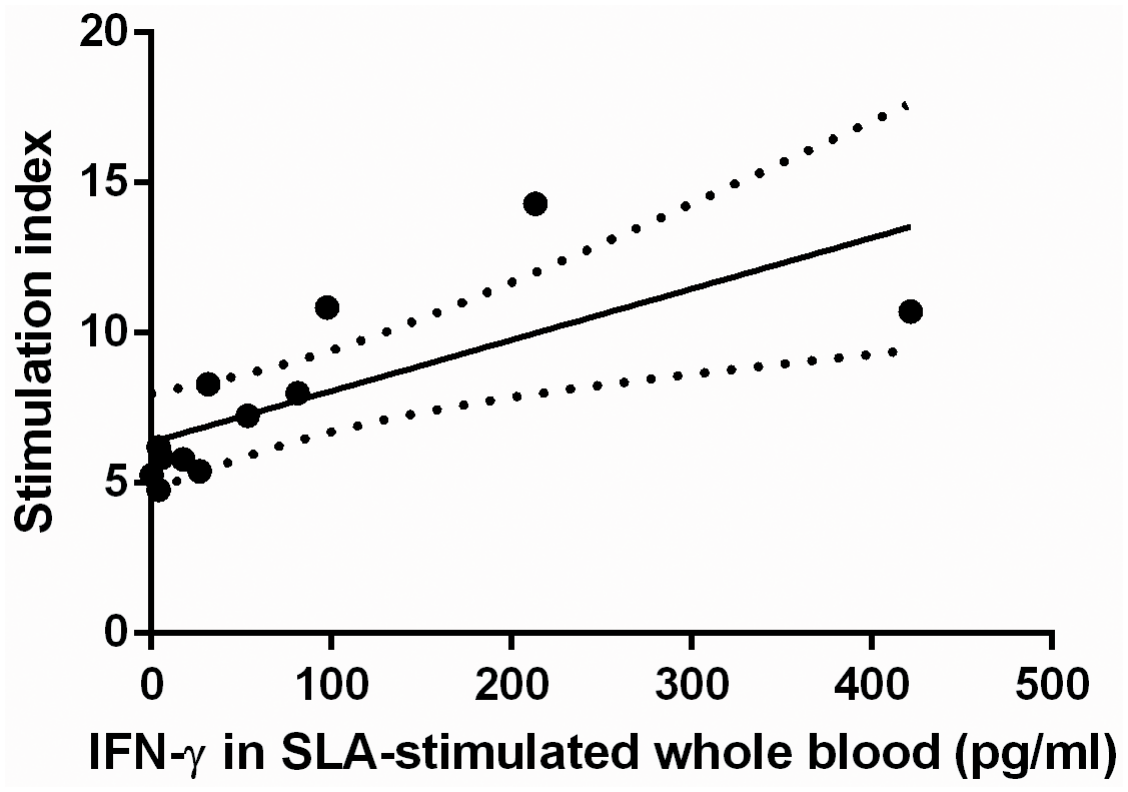
Linear regression of stimulation index and IFN-γ in plasma from SLA-stimulated whole blood plasma from NVL_lp+_ subjects. A significant correlation was detected between these variables (r = 0.881, P = 0.002).

Interestingly, in the NVL_lp+_ subjects, a strong correlation was found between the lymphoproliferative capacity of SLA-stimulated PBMCs and the production of IFN-γ in SLA-stimulated whole blood (r = 0.881, P = 0.0002) (Fig 6).

PHAM stimulation of whole blood led to similar levels of cytokine production in all subjects, with the exception of TNF-α, which was higher in the CVL subjects than in the NVL_lp+_ subjects (P = 0.0061) (Table 2), and higher in the NVL_lp+_ subjects than in the NVL_lp-_ subjects (P = 0.0225).

**Table 2:** Cytokine and granzyme B production in whole blood plasma in response to PHAM stimulation for 24 h. The IFN-γ, granzyme B, TNF-α, IL-10 and IL-2 concentrations (pg/ml) were measured for 12 NVL_lp+_ subjects, 7 NVL_lp-_ subjects, and 5 CVL subjects.

SOT	IFN-γ (pg/ml)	TNF-α (pg/ml)	IL-10 (pg/ml)	Granzyme B (pg/ml)	IL-2 (pg/ml)
	Mean SD	Mean SD	Mean SD	Mean SD	Mean SD
NVL_lp+_	458.42 ± 761.54	245.22* ± 309.82	769.90 ± 584.38	1242.02 ± 1518.37	12.61 ± 43.70
CVL	262.90 ± 464.70	2237.00** ± 3106.00	368.80 ± 267.00	646.90 ± 837.50	0.00 ± 0.00
NVL_lp-_	160.79 ± 183.00	791.00 ± 562.10	372.79 ± 214.90	756.48 ± 545.10	0.00 ± 0.00

Abbreviations:

NVL subjects: Solid organ transplant recipients whom reported no previous episode of VL or compatible symptomology;

NVL_lp+_: NVL subjects who mounted a positive lymphoproliferative response to SLA stimulation;

CVL: Solid organ transplant recipients cured form visceral leishmaniasis;

NVL_lp-_: NVL subjects who did not respond to SLA;

*p<0.05

**p<0.01

## Discussion

The present results show that tests for the cell–mediated immune response are crucial for detecting exposure to *L*. *infantum*; serological tests alone are insufficient. This is especially true when trying to detect evidence of exposure in immunosuppressed individuals, who are at greater risk of developing clinical leishmaniasis after becoming infected, or relapsing after treatment for VL. Few studies have examined the exposure of a large population of SOT recipients living in an area with a high level of endemic leishmaniasis. To our knowledge, this is the first work to study the cell-mediated immunological response to *Leishmania* in such subjects. In early post-transplant patients, parasites have been detected in the bone marrow [15] and liver [6], the consequence of dormant infections. Although, it is unusual to find parasites in the blood of SOT recipients with no symptoms of leishmaniasis, Clemente et al. (2014) reported circulating leishmanial DNA and anti-*Leishmania* antibodies in one out of 50 liver transplant recipients in Brazil [6]. In the present work no subject was detected with leishmanial DNA in the blood. However, *Leishmania*-specific serology was detected in 2 of the 57 NVL subjects (3.50%). In areas with high transmission rates, the tests commonly used to detect exposure are *in vitro* antigen recall experiments and the Leishmanin skin test (LST). These tests show good agreement (98–100%) [16] [17]. However, LST testing cannot be performed in Europe since there is no registered commercial reagent for use with humans. Whole-blood IGRAs, offer alternatives. In fact, in intracellular tuberculosis infections, IGRAs generally have a better detection rate than the tuberculin skin test (TST) [18].

In the present work, supernatants from cultures of SLA-stimulated PBMCs from CVL subjects showed a patent lymphoproliferative response and strong production of IFN-γ and TNF-α. The response of the NVL_lp+_ subjects was very similar. The same responses have been widely reported in immunocompetent cured patients [19, 20] and asymptomatic individuals [21, 22]. The NVL_lp+_ subjects can thus be considered asymptomatic. This shows that, in spite of their drug-induced immunosuppression, some SOT recipients in contact with *Leishmania* can mount a specific Th1 response against the parasite.

One published work has recently suggested the measurement of granzyme B as a new marker for asymptomatic individuals and those clinically cured of VL and cutaneous leishmaniasis [23]. In the present work, granzyme B production strongly increased after SLA stimulation in the NVL_lp+_ and CVL subjects. Further, a correlation was seen between IFN-γ, TNF-α and granzyme B production in supernatants from SLA-stimulated PBMC cultures from NVL_lp+_ subjects. This is the first study to show granzyme B can be used as a marker of an immunological response to *Leishmania* in immunosuppressed individuals.

SLA stimulation of PBMCs from NVL_lp+_ subjects led to significant increases in IL-5, IL-17A and IL-10 production. Correlations between IL-10/IL-5 and IFN-γ have previously been reported in asymptomatic subjects [21, 24]. Further, IL-17A production has been reported to increase in exposed but healthy, resistant subjects who did not develop VL [25]. The relationship between IL-17A and protection against leishmaniasis in SOT recipients deserves further study.

Proof of the accuracy of IGRA tests for screening exposed individuals in areas with endemic leishmaniasis already exists [26–28]. However, clinical experience with whole blood stimulation assays to detect *Leishmania* infection is limited. In fact, this is the first study to assess the predictive capacity of this assay in a large cohort of SOT recipients. Interestingly, a strong, positive correlation was detected between the *in vitro* SLA lymphoproliferative SI and IFN-γ production in SLA-stimulated whole blood from NVL_lp+_ subjects, showing that cytokine assays can be used to test immunological status. In addition, SLA-stimulated whole blood from these NVL_lp+_ subjects showed increases in IFN-γ, TNF-α and IL-2. It has been reported that whole-blood cells from the majority of patients with active VL, and those cured of it, as well as 24% of healthy controls, produce significantly elevated levels of IFN-γ [26]. An increase in TNF-α and an absence of IL-10 after SLA stimulation in healthy controls has also been noted [28]. However, this is the first time that differences in IL-2 between NVL_lp+_ and NVL_lp-_ subjects have been reported. Certain combinations of cytokines may therefore serve as markers of exposure and of cure. Currently, donor screening for leishmaniasis is not performed; the present results suggests that both organ donors and recipients should be screened for this infection.

The present results confirm the usefulness of the whole blood stimulation assay and of IFN-γ/ TNF-α analysis for following up SOT recipients treated for VL; they could help confirm cure and prevent relapses. Indeed, the tests performed detected one recipient who had recently become infected who developed active leishmaniasis in the following days.

### Conclusions

The present results highlight the need to use tests that detect the cell-mediated immune response when screening for asymptomatic subjects in areas with endemic leishmaniasis. They also show that PBMC cultures and/or whole blood assays can be used to search for leukocyte cytokine production as a marker of infection. The production of IFN-γ, TNF-α, granzyme B, IL-5 and IL-10 by SLA-stimulated PBMCs, and of IFN-γ, TNF-α and IL-2 by SLA-stimulated whole blood, could be used to indicate exposure to leishmaniasis, especially for patients subjected to induced immunosuppression. The combination of serological and cell-based tests could help determine the true size of the epidemic of leishmaniasis affecting Fuenlabrada, and indeed of those affecting other areas.

## Supporting Information

S1 Checklist STARD checklist. (DOC)

S1 Table IL-17A, IL-5 and IL-4 production in whole blood plasma in response to SLA stimulation for 24 h. Concentration of cytokines (pg/ml) were measured for 12 NVL_lp+_ subjects, 7 NVL_lp-_ subjects, and 5 CVL subjects.
(DOCX)

S1 Fig Flow chart of the SOT subjects included in the study. (TIF)
