# Supplementary material for: Cytokine Release Assays as Tests for Exposure to Leishmania, and for Confirming Cure from Leishmaniasis, in Solid Organ Transplant Recipients
Source: PLoS Negl Trop Dis. 2015 Oct 23;9(10):e0004179. doi: 10.1371/journal.pntd.0004179 (PMC4619795; doi:10.1371/journal.pntd.0004179)
Supplement: S1 Table — Concentration of cytokines (pg/ml) were measured for 12 NVLlp+ subjects, 7 NVLlp- subjects, and 5 CVL subjects. (DOCX) [file pntd.0004179.s002.docx]

| SOT | IL-17A (pg/ml) | | | IL-5 (pg/ml) | | | IL-4 (pg/ml) | | |
| --- | --- | --- | --- | --- | --- | --- | --- | --- | --- |
|  | Mean |  | SD | Mean |  | SD | Mean |  | SD |
| NVL_lp+_ | 0.00 | ± | 0.00 | 0.02 | ± | 0.07 | 0.05 | ± | 0.16 |
| CVL | 0.00 | ± | 0.00 | 23.05 | ± | 51.53 | 0.00 | ± | 0.00 |
| NVL_lp-_ | 0.00 | ± | 0.00 | 0.00 | ± | 0.00 | 0.05 | ± | 0.13 |
