# Supplementary figures and images for: Cytokine Release Assays as Tests for Exposure to Leishmania, and for Confirming Cure from Leishmaniasis, in Solid Organ Transplant Recipients
Source: PLoS Negl Trop Dis. 2015 Oct 23;9(10):e0004179. doi: 10.1371/journal.pntd.0004179 (PMC4619795; doi:10.1371/journal.pntd.0004179)

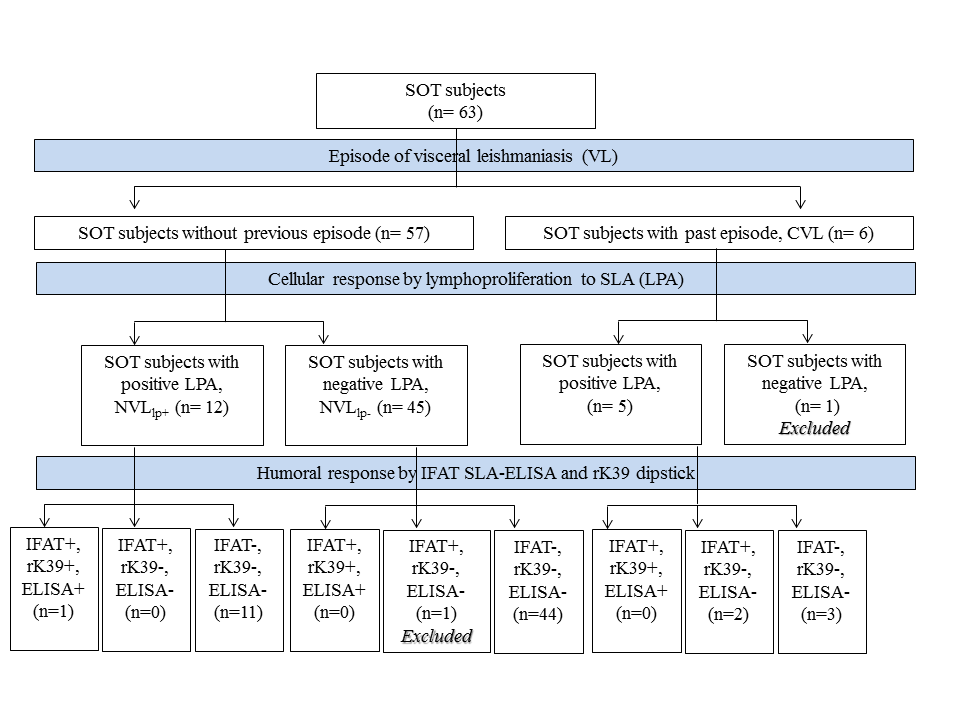

Supplement: S1 Fig — (TIF) [file pntd.0004179.s003.tif]
